# Burden of Respiratory Disease in Pediatric Intensive Care Unit: Experience from a PICU of a Tertiary Care Center in Pakistan

**DOI:** 10.1155/2024/6704727

**Published:** 2024-08-06

**Authors:** Sidra Ishaque, Nazia Bibi, Zaiba Shafik Dawood, Janeeta Hamid, Quratulain Maha, Syeda Asma Sherazi, Ali Faisal Saleem, Qalab Abbas, Naveed Ur Rehman Siddiqui, Anwar Ul Haque

**Affiliations:** ^1^ Department of Pediatrics The Aga Khan University Hospital, Karachi, Pakistan; ^2^ Medical College The Aga Khan University Hospital, Karachi, Pakistan; ^3^ Department of Pediatrics Liaquat National Hospital, Karachi, Pakistan

## Abstract

**Introduction:**

We aimed to determine the burden of respiratory disease by examining clinical profiles and associated predictors of morbidity and mortality of patients admitted to a Pediatric Intensive Care Unit (PICU) in Pakistan, a resource limited country. We also stratified the respiratory diseases as defined by the Pediatric Advanced Life Support (PALS) Classification.

**Methods:**

A retrospective study was conducted on children aged 1 month to 18 years who were diagnosed with respiratory illness at the PICU in a tertiary hospital in Karachi, Pakistan. Demographics, essential clinical details including immunization status, and the outcome in terms of mortality or survival were recorded. Predictors of mortality and morbidity including prolonged intubation and mechanical ventilation in the PICU were analyzed using the chi-square test or Fischer's exact test as appropriate.

**Results:**

279 (63.8% male; median age 9 months, IQR 4–36 months) patients were evaluated of which 44.2% were malnourished and 23.3% were incompletely immunized. The median length of stay in the PICU was 3 days (IQR 2–5 days). Pneumonia was the principal diagnosis in 170 patients (62%) and accounted for most deaths. 76/279 (27.2%) were ventilated, and 67/279(24.0%) needed inotropic support. A high Pediatric Risk of Mortality (PRISM) III score, pneumothorax, and lower airway disease were significantly associated with ventilation support. The mortality rate of patients was 14.3%. Predictors of mortality were a high PRISM III score (OR 1.179; 95% CI 1.024–1.358, *P*=0.022) and a positive blood culture (OR 4.305; 95% CI 1.062-17.448, *P*=0.041).

**Conclusion:**

Pneumonia is a significant contributor of respiratory diseases in the PICU in Pakistan and is the leading cause of morbidity and mortality. A high PRISM III score, pneumothorax, and lower airway disease were predictors for ventilation support. A high PRISM III score and a positive blood culture were predictors of patient mortality in our study.

## 1. Introduction

Respiratory disease is a condition of abnormal respiratory rate and effort. Respiratory disease is responsible for over 10 million admissions and deaths in children younger than 5 years of age. It is the leading cause of death in children in low-middle income countries (LMICs) [1]. The number of deaths attributed to respiratory disease in low- and middle-income countries is 4 million [2]. Additionally, lower respiratory tract infections are a leading cause of death in children in Pakistan [3, 4]. A global burden of disease report concluded that Pakistan ranked seventh highest in burden of respiratory disease and had the highest burden of tuberculosis globally [5].

Pediatric respiratory disease leads to increased financial and nonfinancial burden on families of affected children which in turn especially affects LMICs that already have few resources to support such children [6]. This is particularly stressful in LMICs like Pakistan where most patients pay out of pocket with limited access to insurance and support from the government. Moreover, it results in lots of time spent in the hospital leading to disruption of work and the normal lives of families [7, 8]. Respiratory diseases such as pneumonia are also associated with several complications such as pneumothorax and acute respiratory distress syndrome (ARDS) [9], which increase morbidity and reduce patient prognosis. There have been many studies done in pediatric patients admitted to PICU in LMICs such as India [10] and China [11] that have shown the mortality rate due to respiratory disease ranging from 12% to 30% and risk factors being age less than 1 year, malnutrition, and inotropic support. So far, there have been only few studies done in Pakistan which look at the predictors of mortality in the PICU. One study in Pakistan looked at all admissions in the PICU and assessed the risk factors of mortality [12], and another study looked at the outcomes of patients mechanically ventilated but only comprised 60 patients with respiratory disease [13]. To the best of our knowledge, this is the largest study from a Pediatric Critical Care Unit in Pakistan evaluating the clinical profile, management, and predictors of mortality in patients admitted with respiratory disease in the PICU.

We additionally stratified the results of our study based on Pediatric Advanced Life Support (PALS) which refers to the set of guidelines through which health care providers assess and care for infants and children in the critical care and intensive care units [14]. According to PALS, respiratory disease is classified into four types including upper air way, lower airway obstruction, lung tissue disease, and disordered control of breathing [15]. We stratified our results based on these guidelines to assess the number and outcomes of patients belonging to each specific group.

With the growing burden of respiratory disease in LMICs and the scarcity of data from regions like Pakistan, it is important to assess the pattern of presentation, management, and outcomes of respiratory diseases in the PICU stratified by the PALS criteria in order to identify disease presentations which are associated with poor outcomes and direct resources towards them. Hence, we sought to determine the burden of respiratory diseases, associated risk factors, and predictors of morbidity and mortality amongst patients admitted to the PICU in a single center in Karachi, Pakistan, and stratify the results as defined by the PALS classification.

## 2. Materials and Methods

This study was conducted at the PICU of Aga Khan University Hospital (AKUH), a tertiary care hospital in Karachi, Pakistan. The PICU is an eleven-bedded closed multidisciplinary unit with 450–500 admissions annually. It is staffed by a senior resident and a fellow 24 hours a day, supervised by four trained pediatric intensivists. The patient-to-nurse ratio is 1 : 1, and all physicians and nurses are PALS certified. The PICU has all the facilities available including means of noninvasive ventilation such as high flow nasal cannula, BIPAP, and high-frequency oscillatory ventilation (HFOV). Extracorporeal membranous oxygenation is unavailable. Patients are admitted from the emergency department (ED) directly after presentation or after being referred to our center from nearby or remote regional hospitals with occasional travel time as long as 12 hours.

The PICU of AKUH follows standard management guidelines for different diseases, such as PALS guidelines for shock management and Pediatric Acute Lung Injury Consensus Conference (PALICC) guidelines for pediatric acute respiratory distress management, among others [16].

The Ethical Review Committee of AKUH, after having reviewed the study aims, provided a waiver from approval-waiver number 2020-3510-8668. Data were then collected from 1^st^ January 2021 to 31^st^ December 2022. We included all pediatric patients (age from 1 month to 18 years) admitted to the PICU for acute respiratory disease or cardiorespiratory disease. We excluded patients admitted for nonrespiratory disease, postoperative cases, post-trauma cases (developing respiratory distress and complications during PICU stay), and patients with tachypnea due to acidosis. Patients were identified through the hospital's health information management system using the International Statistical Classification of Diseases and Related Health Problems, 10th revision (ICD-10) coding for mortality and were confirmed by cross-referencing PICU logbooks and the hospital's mortality meeting records.

We grouped the etiology of patients' respiratory disease into four categories as defined by PALS: upper airway disease, lower airway disease, parenchymal disease, and disordered control of breathing. We also involved patients who had respiratory disease stemming from a cardiac problem, and we labelled it cardiorespiratory disease.

Patients who developed either hypoxemia with pO2 less than 60 mm Hg, or hypercapnia with pCO2 greater than 55 mm, and patients requiring invasive mechanical ventilation or escalation of noninvasive ventilation with hemodynamic instability were considered as having respiratory failure in this study [17]. Acute respiratory distress was diagnosed using Pediatric Acute Lung Injury Consensus Conference (PALICC) criteria [18]. Acute onset, new infiltrates on chest imaging that displayed acute parenchymal disease, and edema not explained by cardiac failure or fluid overload were considered as ARDS by this study.

The demographic data (age, gender, duration of stay in PICU, and hospital stay), essential clinical details, diagnosis at admission, risk factors for mortality, laboratory and radiographic reports, details of mechanical ventilation, treatment details, course in the PICU, and the hospital and the outcome were recorded in a specially designed proforma. The outcome was measured in terms of mortality or survival.

Data pertaining to vaccination and malnutrition were also recorded. Age, height, and weight were used to plot the WHO standardized growth charts. Patients who were under the fifth percentile were classified as malnourished. Patients were classified as completely or partially unimmunized based on a standard Expanded Programme on Immunization (EPI) vaccine schedule from Pakistan [19]. The EPI schedule vaccinates children against tuberculosis, poliomyelitis, diphtheria, tetanus, pertussis, hepatitis B, haemophilus influenza type b, and measles.

Predictors of mortality assessed including the severity of disease by Pediatric Risk of Mortality III score‐ PRISM III score [20], type of disease or diagnosis, need for mechanical ventilation and duration, and length of stay were analyzed using the chi-square test.

### 2.1. Statistical Analysis

All the data were entered into the Statistical Package for Social Sciences (SPSS) version 23 and analyzed. The respiratory diseases were stratified further as mentioned in PALS. Association between qualitative variables, e.g., severity of respiratory disease at the time of admission and length of mechanical ventilation and total duration of hospital stay, was assessed using chi-square or Fischer's exact test as appropriate. The percentage of various etiologies, diagnosis of respiratory diseases, and diagnosis‐wise mortality were determined. *P* value of less than 0.05 was taken as significant.

## 3. Results

The study examined 279 pediatric patients (age from 1 month to 18 years); the median age of patients was 9 months (IQR 4–36 months), and majority of them were male (178 patients; 63.8%). Of the study population, 151/279 (54.1%) were less than 1 year of age; almost a quarter of patients had incomplete immunization (65; 23.3%) while almost half of patients (122; 44.2%) weighed less than 5^th^ percentile on the growth curve and hence were considered malnourished. Of note, the largest proportion of admission diagnosis was occupied by pneumonia (150 patients; 53.8%) followed by bronchiolitis (42 patients; 15.1%). Specifically, 213/279 (76.9%) of patients had a PRISM III score between 0 and 5; 226/279(81%) of patients had respiratory distress, while 25/279 (19%) had respiratory failure which was diagnosed by looking at the metrics of oxygenation including the oxygen index and oxygen saturation index as is defined by Nadir et al. [21].  Table 1 summarizes the demographics of the study population and risk factors of severe disease.

Of note, fever (81.0%) and cough (77.1%) were the major symptoms, while crepitus (50.5%), tachypnea (37.3%), and decreased air entry (25.8%) were the major signs of disease. Additionally, 127/279 (45.8%) had a normal total leukocyte count (TLC); 254/279 (91.7%) had an abnormal X-ray. Specifically, 30/254 (11.8%) had pleural effusion; 73/254 (28.7%) had atelectasis, 28/254 (11.0%) had consolidation, 16/254 (6.3%) had pulmonary edema, 13/254 (5.12%) had pneumothorax, and 120/254 (47.2%) had other abnormalities such as signs of reactive airway disease or asthma including hyperinflated lung fields and peribronchial cuffing, air trapping, and haziness. In addition, 21/279 (7.5%) patients had an abnormal CT scan mainly showing pneumothorax, empyema, subcutaneous emphysema, necrotizing pneumonia, pneumothorax, pleural effusion, or consolidation. Tracheal cultures were positive in 23/279 patients (8.2%) of which 5/23 had MRSA and 4/23 had Acinetobacter and 5/23 had pseudomonas on culture.

Table 2 summarizes the signs, symptoms, and laboratory investigations performed, and Table 1 shows the classification of diagnosis according to the PALS where most (62%) of the patients had parenchymal disease followed by lower airway disease (24%).

Of the study population, 76 patients (27.2%) needed ventilation, with median duration being 4 days (IQR 2-8 days). In addition, almost a quarter of patients (67 patients, 24%) needed inotropic support with a mean vasoactive inotropic support (VIS) score of 8.0. The median length of stay in the PICU was 3 days (IQR 2–5 days) and in the hospital was 6 days (IQR 4–10 days). Of note, intubated patients had higher length of stay compared to nonintubated patients (10.9 vs. 6.8 days, respectively, *P* < 0.001). Only 3/279 patients underwent tracheostomy. One was due to Guillan Barre Syndrome in the same admission, the other was due to pyknodysostosis suspected in the same admission, and the third one was due to extradural hematoma in a previous admission.  Table 3 shows the management done in PICU and the outcomes of patients.

On multivariate analysis, patients with a higher PRISM III score (OR, 1.164; 95% CI, 1.079–1.254), those with respiratory failure (OR, 0.493; 95% CI, 1.022–6.081), pneumothorax (OR, 7.455; 95% CI, 1.582–35.125), and lower airway (OR, 0.225; 95% CI, 0.070–0.723) disease were significantly more likely to be intubated. Additionally, a high PRISM III score (OR, 1.345; 95% CI, 1.170–1.548), respiratory failure (OR, 41.134; 95% CI, 9.061-186-743), pneumothorax (OR, 74.688; 95% CI, 3.091–1804.413), and atelectasis (OR, 8.026; 95% CI, 2.479–25.988) were significant predictors of inotrope use (Tables 4 and 5, respectively).

In this study, 40 patients (14.3%) did not survive and on multivariable analysis, the variables found to be significantly associated with mortality were of a higher PRISM III score (OR, 1.179; 95% CI, 1.024-1.358; *p*=0.041) and positive blood culture (OR 4.305; 95% CI, 1.062–17.448; *p*=0.022).  Table 6 shows the multivariate analysis of risk factors associated with mortality.

Table 7 shows selected demographic characteristics, risk factors, PICU management, and outcome of patients based on PALS classification where a significantly higher percentage of patients with lower airway disease were malnourished (*p* value = 0.016) and had respiratory failure (*p* value <0.001). Patients with disordered control of breathing also had the highest PRISM III score (*p* value <0.001). A significantly higher portion of cardiac patients required ventilation (*p* value = 0.001) and inotropic support (*p* value <0.001).

Tables 2 and 3 show the other difference in demographics, signs, and symptoms of patients based on PALS classification where a significantly higher portion of patients with lower respiratory disease presented with cough (*p* value = 0.001). A significantly higher portion of patients with disordered control of breathing had fever (*p* value <0.001). A significantly higher proportion of patients with upper airway disease presented with decreased air entry (*p* value = 0.033).

## 4. Discussion

Pediatric respiratory disease is a significant cause of morbidity and mortality [22]. Additionally, it causes a great financial and resource restraint in LMICs [1], where admission in the PICU is very costly especially since respiratory diseases cause prolonged length of stay and expenses are often paid out of pocket [23]. This highlights the morbidity, mortality, and financial burden of pediatric respiratory diseases. As such, it is important to assess factors associated with admission to PICU and those contributing towards prolonged mechanical ventilation and inotropic support in LMICs such as Pakistan. This is the largest study to be conducted in Pakistan that assesses the burden of respiratory diseases in children admitted to the PICU stratified by the PALS criteria. The demographics of patients admitted to the PICU in this study, most patients being males and infants, is similar to other studies conducted in the PICU in India [10], South Africa [16], and Istanbul [21]. Infants who present to the PICU have a higher mortality rate due to increased requirement of invasive mechanical ventilation [10]. This means that the PICU needs to be well supplied with resources to specifically handle infants including the availability of better equipment and establishment of well-trained personnel which could greatly help reduce mortality [24].

Almost a quarter of patients in this cohort were incompletely immunized and almost half were malnourished. This is similar to previous studies conducted in India [10, 25], while these rates are significantly higher than previous studies in South Africa [16] and across developed countries [26]. Incomplete immunization and malnutrition are major causes of mortality in children with respiratory disease in the PICU [10]. So, with increased presentation of such cases in India and Pakistan, there is increased importance of the work done by community health workers to ensure complete immunization and adequate nutrition. This is especially the case with postmeasles pneumonia (PMP) which occupied over 20% of pneumonia cases in our study. PMP presents as a complication of pneumonia in unimmunized children and is associated with a higher rate of complications leading to increased morbidity [9]. Hence, complete immunization would prevent such cases and reduce the morbidity attached. Malnutrition increases the susceptibility to infections which could be linked to the reduced production of inflammatory cytokines [27]. This could also be the reason why a significantly higher proportion of malnourished patients in this study had lower respiratory disease which is similar to a nationwide study on the global etiology on LRTI [28].

More than half of patients in this cohort presented with parenchymal diseases which is similar to the study conducted in India [10] and Singapore [29]. Pneumonia and ARDS on pneumonia contributed to 71% and 50% of mortality, respectively. Similar results have been reported in previous studies [10, 30]. Hence, it is important to provide greater care to children presenting with pneumonia in the PICU in order to prevent the increase of severity of disease and hence reduce mortality.

Patients with disordered control of breathing had the highest PRISM III score and also reported to have fever more frequently. Fever and disordered control of breathing could be related to the central control of both temperature and respiration, and hence, a central defect in one mechanism could lead to a disruption in another. Such patients may also experience respiratory failure, and this may worsen PRISM III scores. However, these concepts still need to be investigated further. Hence, further studies on the association between disordered control of breathing, a high PRISM III score, and fever are warranted.

Respiratory diseases stem as the second most common reason for mechanical ventilation [20]. Moreover, prolonged mechanical ventilation poses a great impact on children's physical and mental health [31]. At our center, we provide both invasive and noninvasive respiratory support. Invasive modalities include conventional invasive mechanical ventilation and high-frequency oscillatory ventilation (HFOV). Noninvasive modalities include noninvasive mechanical ventilation (NIMV) and High Flow Nasal Cannula (HFNC). In our study, the median duration of ventilation was 4 days with only 24% of patients requiring intubation. The duration of ventilation was significantly higher than another study conducted in the PICU in Pakistan [20]. These rates are however significantly lower than studies conducted in Singapore [29] and South Africa [16]. The low number of patients requiring ventilation in this study could be attributed to the fact that a majority of patients had fewer comorbidities such as underlying cardiorespiratory diseases compared to previous studies, and hence, their lungs were able to sustain the patients throughout the disease. Additionally, our study found that a higher PRISM III score, respiratory failure, pneumothorax, and lower airway disease were significantly associated with ventilation. Pneumothorax raises invasive ventilation need and prolongs ventilation duration [32]. On the other hand, respiratory failure is a major indication for mechanical ventilation and leads to fatal outcomes in children [33]. A higher PRISM III score has been associated with mortality in a couple of studies [34]. However, the variables used to determine PRISM III score are also variables that predict respiratory failure. An overlap between the two may be a possibility. Hence, more studies are required in order to assess the relationship between the PRISM III score and mechanical ventilation. Lower airway disease has been linked to prolonged mechanical ventilation in a previous study [16]. Hence, patients with LRTI and a high PRISM III score should be monitored for the possibility of mechanical ventilation.

Inotropic support is generally required in children with cardiac and hemodynamic instability. However, high VIS is adversely associated with poor outcomes such as mortality, delayed extubation, and prolonged LOS [35]. In our study, inotropic support was required in 26% of patients which is similar to that of India [36], while it is significantly higher than the study conducted in South Africa [16]. This could be due to the improved care in the PICU in South Africa compared to that of India and Pakistan. Moreover, the factors significantly associated with inotropic support were a high PRISM III score, pneumothorax, and atelectasis. A high PRISM III score has been linked to inotrope support in a study in Iran [37]. However, literature on the relationship between high inotropic support and pneumothorax is limited.

The mortality rate of patients in this study (14.3%) is similar to that of Istanbul and Singapore [21, 29]. The results of our study additionally show that a higher PRISM III score and positive blood culture are significantly associated with mortality. This is similar to the results of the studies in India and Istanbul [10, 21]. PRISM III score is a well-known tool to predict prognosis of patients in the PICU where Bellad et al. showed that with every increase of the PRISM III score by 1, the odds of mortality increase by 36% making the PRISM III score highly sensitive in predicting mortality [38]. This shows the concordance of our results with previous studies and also highlights the need to calculate the PRISM III score to predict patients requiring urgent care and to direct the allocation of resources accordingly.

Our study also shows that a positive blood culture is significantly associated with mortality. This is congruent with a previous study which showed that patients with sepsis with a negative blood culture have a lower mortality than those with a positive culture [39]. However, data for patients admitted with pneumonia blood culture are low yield and not routinely collected [40]. A majority of patients included in our study presented with sepsis, septic shock, and multiorgan dysfunction. In these cases, positive blood cultures helped identify the causative microorganism and make evidence-based decisions for antibiotic stewardship. In isolation, without concomitant signs of bacteremia and septicemia, positive blood cultures have limited use. So far, very few studies have been conducted that evaluate the prognosis of children admitted with pneumonia with a positive blood culture. Hence, more studies are required in order to evaluate the effect of positive blood culture on the outcome of patients admitted with pneumonia.

A significant portion of our data collection period coincided with the second wave of the COVID-19 pandemic in Pakistan, with rising number of pediatric hospitalizations due to Alpha, Delta, and Omicron variants [41]. Therefore, we find it important to comment on the impact of the pandemic on our setup. The PICU at Aga Khan University consists of an eleven bedded facility equipped with state-of-the-art ventilators, advanced monitoring systems, and two negative isolation rooms. At the height of the pandemic, we experienced challenges in healthcare delivery, as the available ventilators in the PICU were overwhelmed. Furthermore, COVID-19 pneumonia proved challenging to treat, particularly in the presence of comorbidities and multisystem inflammatory syndrome. Pediatric patients who develop COVID-19 pneumonia and have comorbid conditions are at a greater risk of mortality and require additional attention and monitoring [42]. However, despite these challenges, pandemic preparedness after the first wave allowed us to manage and treat patients without significant delay. This may partially be attributed to the decreased PICU admissions due to non-COVID-19 cases. A study analyzing PICU admission in Pakistan pre- and postpandemic found that PICU admissions were reduced by 23–44% when compared to the previous years [43]. This study proposed the explanation that the global lockdown, closure of schools and recreational activities, social distancing, and use of masks led to low bacterial and viral illness transmission. Furthermore, trauma-related PICU admissions also decreased [43].

The major strength of our study is that it is the largest retrospective study conducted in Pakistan. The large sample size helps reduce the chance of errors. We also included patients over a period of two years which enabled us to get a wide range of study samples over a long period of time.

This study should be interpreted considering a few limitations. Firstly, despite this study being the largest retrospective study conducted in Pakistan, it is a single-center study which reduces the strength of our results because risk factors for the disease, PICU, and hospital care are similar for all patients. However, prevalence of disease calculated in our study is similar to previous studies that focused on burden of disease in Karachi's slums, which showed the frequency of pneumonia to be 65% compared to our 62% [44].

Secondly, most patients in this center were from Karachi and most had a similar sociodemographic, thus reducing the generalizability of our results. Hence, more multicenter studies should be conducted assessing the burden of respiratory disease in different centers in Pakistan and the demographics, management, and predictors of mortality compared. More studies should also be conducted looking at the predictors of mortality and stratifying data according to PALS classification. In addition, prospective studies over longer durations and statistical data about positive blood cultures would help identify burden of disease and etiology with greater generalizability.

## 5. Conclusion

This study demonstrates that amongst respiratory diseases, pneumonia has a significantly higher contribution (62%). More than 20% of pediatric patients admitted for respiratory causes in the PICU required ventilatory support and inotropic support and had a LOS in the PICU greater than 7 days. Children with pneumothorax, high PRISM III score, and lower airway disease were more likely to be ventilated. Moreover, a high PRISM III score and positive blood culture were significantly associated with mortality.

## Figures and Tables

**Table 1:** Demographics and risk factors of patients in the PICU due to respiratory disease.

Demographics	*N* = 279	%
Gender		
Male	178	63.8
Female	101	36.2
Age (years)		
Mean age (months)	28.4 ± 43	
<1	151	54.1
1–5	89	31.9
6–10	25	9.0
10–15	11	3.9
>15	2	0.7
Immunization status		
Complete	151	54.1
Partial	60	21.5
Unknown	63	22.6
None	5	1.8
Malnourished		
Yes	122	44.2
No	154	55.8
History of TB contact		
Yes	2	0.7
No	277	99.3
PALS classification		
Upper airway disease	12	4.4
Lower airway disease	67	24.4
Parenchymal disease	170	61.8
Disordered control of breathing	22	8.0
Cardiorespiratory disease	4	1.5
Change in consciousness		
No	201	72.0
Yes	78	28.0
Severity		
Respiratory failure	53	19.0
Respiratory distress	226	81.0
PRISM score group		
0–5	213	76.9
6–10	37	13.4
11–15	16	5.8
>15	11	4.0

This table shows the demographic details and risk factors of patients admitted to the PICU with respiratory disease. PICU: Pediatric Intensive Care Unit; PRISM: Pediatric Risk of Mortality III score; PALS: Pediatric Advanced Life Support.

**Table 2:** Signs and symptoms of respiratory disease in patients in the PICU.

Variables	*N* = 279 (%)
Fever	226 (81.0)
Cough	215 (77.1)
Crepitus	141 (50.5)
Tachypnea	104 (37.3)
Decreased air entry	72 (25.8)
Tachycardia	55 (19.7)
Desaturation	32 (11.5)
Rhonchi	7 (2.5)
Cyanosis	11 (3.9)
Stridor	7 (2.5)
TLC	
High	124 (44.8)
Low	18 (6.5)
Normal	127 (45.8)
Not done	8 (2.9)
Blood culture	
Positive	49 (17.7)
Negative	207 (74.7)
Not done	21 (7.6)
Chest X-ray	
Abnormal	254 (91.7)
Normal	17 (6.1)
Not done	6 (2.2)
CT chest	
Abnormal	11
Normal	0 (0)
Not done	234 (91.8)
Tracheal culture	
Positive	24 (9.3)
Negative	37 (14.3)
Not done	198 (76.4)

This table shows the signs, symptoms, and lab tests of patients admitted to the PICU with respiratory disease.

**Table 3:** Management and outcomes of patients in the PICU with respiratory disease.

Management/outcomes	*N* (%)/median (IQR)
Ventilation need	76 (27.2)
Ventilation duration (days)	4 (2–8)
Inotrope need	67 (24.1)
Tracheostomy	3 (1.1)
Pigtail insertion	17 (6.1)
LOS in PICU (days)	3 (2–5)
LOS in hospital (days)	6 (4–10)
Mortality	40 (14.3)

This table shows the management and outcomes of patients admitted to the PICU with respiratory disease. VIS: vasoactive inotropic score; LOS: length of stay; PICU: Pediatric Intensive Care Unit.

**Table 4:** Multivariate analysis of factors associated with ventilation need.

Variables	*P* value	OR (95% CI)
GCS	0.359	0.939 (0.822–1.074)
PRISM score	<0.001	1.164 (1.079–1.254)
Respiratory failure	0.045	2.493 (1.022–6.081)
Pleural effusion	0.277	1.738 (0.641–4.713)
Pulmonary edema	0.362	1.825 (0.501–6.651)
Pneumothorax	0.011	7.455 (1.582–35.125)
Lower airway disease	0.012	0.225 (0.070–0.723)

This table shows the multivariate analysis of factors associated with ventilation need. PRISM: Pediatric Risk of Mortality Score; GCS: Glasgow Coma Scale.

**Table 5:** Factors associated with inotrope need.

Variables	*P* value	OR (95% CI)
Immunization		
Complete	Ref	—
Partial	0.218	0.150 (0.07–3.066)
None	0.400	0.263 (0.012–5.894)
TLC		
Normal	Ref	—
High	0.387	1.645 (0.533–5.079)
Low	0.950	1.121 (0.031–40.944)
GCS	0.545	0.936 (0.755–1.160)
PRISM score	<0.001	1.345 (1.170–1.548)
Severity		
Respiratory distress	Ref	—
Respiratory failure	<0.001	41.134 (9.061-186-743)
Blood culture		
Negative	Ref	—
Positive	0.058	3.281 (0.958–11.233)
Pleural effusion	0.171	3.115 (0.611–15.870)
Atelectasis	0.001	8.026 (2.479–25.988)
Pulmonary edema	0.850	0.812 (0.094–7.017)
Pneumothorax	0.008	74.688 (3.091–1804.413)
Lower airway disease	0.310	2.133 (0.494–9.216)

This table shows the factors associated with inotrope need. Ref: reference; PRISM: Pediatric Risk of Mortality Score; GCS: Glasgow Coma Scale.

**Table 6:** Multivariate analysis of factors associated with mortality.

Variables	*P* value	Odds ratio (95% CI)
Gender	0.202	2.363 (0.630–8.861)
Change in consciousness	0.190	1.857 (0.590–5.847)
PRISM score	0.022	1.179 (1.024–1.358)
Severity	0.812	1.215 (0.244–6.053)
Pneumothorax	0.690	1.907 (0.080–45.552)
Ventilation need	0.724	4.203 (0.030–11.546)
Inotrope need	0.131	2.949 (0.724–12.009)
Pigtail insertion	0.183	5.290 (0.455–61.500)
LOS in PICU greater than 5 days	0.807	3.197 (0.390–26.197)
Ventilation greater than 5 days	0.323	3.707 (0.275–49.910)
Blood culture	0.041	4.305 (1.062–17.448)
Lower airway disease	0.570	0.561 (0.076–4.125)
Disordered control of breathing	0.772	1.336 (0.188–9.523)

This table shows the multivariate analysis of factors associated with mortality. PRISM: Pediatric Risk of Mortality; LOS: length of stay; PICU: Pediatric Intensive Care Unit.

**Table 7:** Selected demographic characteristics, risk factors, management, and outcomes of patients in the PICU based on PALS classification.

Characteristics	Upper airway disease	Lower airway disease	Parenchymal disease	Disordered control of breathing	Cardiorespiratory disease	*P* value
Malnourished						
Yes	4 (33.3%)	18 (27.7%)	85 (50.0%)	11 (52.4%)	3 (75.0%)	0.016
No	8 (66.7%)	47 (72.3%)	85 (50.0%)	10 (47.6%)	1 (25.0%)	
Change in consciousness						
Yes	4 (33.3%)	6 (9.0%)	47 (27.6%)	18 (81.8%)	3 (75.0%)	<0.001
No	8 (66.7%)	61 (91.0%)	123 (72.4%)	4 (18.2%)	1 (25.0%)	
Mean PRISM score	1.18 ± 2.44	1.67 ± 3.89	3.05 ± 4.96	8.55 ± 7.13	5.00 ± 1.73	<0.001
Severity						
Respiratory distress	1 (8.3%)	6 (9.0%)	27 (15.9%)	15 (68.2%)	3 (75.0%)	<0.001
Respiratory failure	11 (91.7%)	61 (91.0%)	143 (84.1%)	7 (31.8%)	1 (25.0%)	
Ventilation need						
Yes	6 (50%)	10 (14.9%)	44 (25.4%)	13 (59.1%)	3 (75.0%)	0.001
No	6 (50%)	57 (85.1%)	127 (74.7%)	9 (40.9%)	1 (25.0%)	
Mean ventilation duration (days)	5	6	5	7	8	0.724
Inotrope need						
Yes	3 (25%)	4 (6%)	45 (26.6%)	10 (54.5%)	4 (100.0%)	<0.001
No	9 (75%)	63 (94.0%)	124 (73.4%)	12 (54.5%)	0 (0.0%)	
VIS	4.7 ± 4.72	8.8 ± 3.89	8.7 ± 6.34	8.3 ± 6.89	4 ± 1.73	0.644
Pigtail insertion						
No	11 (91.7%)	67 (100%)	157 (92.4%)	20 (90.9%)	4 (100%)	0.200
Yes	1 (8.3%)	0 (0%)	13 (7.6%)	2 (9.1%)	0 (0%)	
Mean LOS in PICU	6.11 ± 3.51	3.64 ± 3.92	4.42 ± 4.13	5.57 ± 5.19	7.50 ± 7.78	0.193

This table shows the selected demographic characteristics, risk factors, management, and outcomes of patients in the PICU based on PALS classification. VIS: vasoactive inotropic score; LOS: length of stay; PICU: Pediatric Intensive Care Unit; PRISM: Pediatric Risk of Mortality III score; PICU: Pediatric Intensive Care Unit.

## Data Availability

The clinical data used to support the findings of this study are restricted by the Ethical Review Committee at Aga Khan University, Pakistan, in order to protect patient privacy. Data are available from Dr Sidra Ishaque (sidra.ishaque@aku.edu) for researchers meeting the criteria for access to confidential data.

## Additional Points

*Highlights*. (1) First study to be conducted in Pakistan to evaluate the burden of respiratory illness in the Pediatric Intensive Care Unit (PICU) stratified by Pediatric Advanced Life Support (PALS). (2) Almost half of the patients were malnourished, and a quarter of them were unimmunized. (3) A high PRISM III score and a positive blood culture were significantly associated with patient mortality.
